# A numerical study of a lifted $\hbox {H}_2/\hbox {N}_2$ flame excited by an axial and flapping forcing

**DOI:** 10.1038/s41598-022-06740-4

**Published:** 2022-02-17

**Authors:** Artur Tyliszczak, Agnieszka Wawrzak

**Affiliations:** Faculty of Mechanical Engineering and Computer Science, Czestochowa University of Technology, Al. Armii Krajowej 21, 41-201 Czestochowa, Poland

**Keywords:** Energy science and technology, Engineering

## Abstract

The large eddy simulation method combined with the Eulerian stochastic field approach has been used to study excited lifted hydrogen flames in a stream of hot co-flow in a configuration closely corresponding to the so-called Cabra flame. The excitation is obtained by adding to an inlet velocity profile three types of forcing [(1) axial; (2) flapping; (3) combination of both] with the amplitude of 15% of the fuel jet velocity and the frequency corresponding to the Strouhal numbers $St=0.30,\, 0.45,\, 0.60\, \,\text {and}\, \,0.75$. It is shown that such a type of forcing significantly changes the lift-off height ($L_h$) of the flame and its global shape, resulting in the flame occupying a large volume or the flame, which transforms from the circular one into a quasi-planar one. Both the $L_h$ and size of the flames were found to be a function of the type of forcing and its frequency. The minimum value of $L_h$ has been found for the case when the axial and flapping forcing were combined and acted at the forcing frequency close to the preferred one in the non-excited configuration. The impact of the flapping forcing manifested through a widening of the flame in the flapping direction. It was shown that the excitation increases the level of temperature fluctuations caused by an intensified mixing process. The computational results are validated based on the solutions obtained for the non-excited flame for which experimental data are available.

## Introduction

Intensive research on applications of active flow control methods was initiated by a famous work of Crow and Champagne^1^ concerning a round jet. Applying a low amplitude excitation (forcing) being a sinusoidal function of time they observed that for some range of frequency the jet behaviour significantly changes. The turbulence intensity level expressed in terms of the velocity fluctuations increased, and moreover, its profile along the jet axis was characterised by a distinct local maximum at a distance of approximately eight nozzle diameter from the nozzle exit. Such behaviour was never reported before and did not occur in natural jets. It turned out that by applying a relatively simple control technique one may change the flow dynamics to the extent incomparably larger to an energy input needed to introduce the excitations. These findings stimulated very extensive analyzes of various excitation types including axial, flapping and helical forcing modes^2–4^, and their combinations. Recent LES (Large Eddy Simulations) and DNS (Direct Numerical Simulations) works of Tyliszczak A.^5,6^ and Gohil et al.^7^ showed that for carefully selected frequencies of combined axial and helical excitation one can obtain multi-armed jets, i.e., the jets characterized by 5, 7 or 11 or even 20 separate branches, closely reminding the blooming jets reported by Reynolds and Parekh^8^. Without doubts, the active flow control methods are superior compared to the passive methods relying on optimization of a flow domain for particular flow regimes. Mainly, because their driving parameters (the excitation amplitude/frequency, spatial distribution, etc.) can be dynamically adapted to changing flow conditions, e.g., increasing/decreasing inlet velocity or temperature. The passive methods do not allow for such freedom, yet, from the point of view of the working costs, they are certainly cheaper as they do not need additional energy to operate.

The findings on the active control techniques quickly translated to combustion science where they have been the focus of interest since the early 1990s^9,10^. Regarding the fundamental problems, attention is very often paid to jet-type flames in which the mechanical or acoustic forcing acts as a source of external excitation. The former is usually introduced by specially designed nozzle tips with magnetic or piezoelectric actuators^11,12^. The acoustic excitation is more often used and is added by loudspeakers mounted upstream of the nozzle exits^13–17^. The influence of this type of excitation on the reduction of pollution emissions in a lean premixed lifted flames and flame stability was demonstrated by Chao et al.^13,14^, among others. Focussing on stability issues, they found that the excitation significantly alters the flame dynamics and can be used as a “tool” suppressing or amplifying the stabilization process. Abdurakipov et al.^15^ demonstrated that in comparison to natural flames the excitation ensures stable combustion regimes and visibly shifts the blow-off limits. Research on an excited lifted non-premixed flame in a hysteresis regime, i.e., when depending on initial conditions a flame can be attached to a nozzle or remain lifted for the same fuel velocity, was performed by Demare and Baillot^16,17^. It was shown that by changing the amplitude and frequency of excitation one can enhance the combustion process or produce large fluctuations, and thus, weaken the flame stability. Kozlov et al.^18^ analyzed micro-flames in the field of transverse sound waves. They found that the excitation can flatten the round flames and transform them into nearly plane flames. Surprisingly, for particular forcing frequencies the excitation led to a splitting of the flame into two separate branches in a very similar way as observed for bifurcating non-reacting, constant density jets^8^. More recently, the occurrence of the bifurcating phenomenon in flames was reported in numerical studies of Tyliszczak^19^ focused on a low Reynolds number hydrogen flame. Application of only the flapping excitation caused the flame to change its initial circular shape into the planar one with two co-existing separate arms. Moreover, for some range of the excitation frequency, a triple-flame occurred.

As discussed above, the excitation can alter the flame dynamics and influence pollution emission. In the present work we apply the LES method in the combination with the Eulerian Stochastic Field (ESF) approach^21^ for the combustion modelling, and focus on the global impact of the excitation on the flame by applying three different excitation types (axial, flapping, axial plus flapping) with different forcing frequencies. Although the basic flow configuration closely corresponds to a well known Cabra flame^20^ at the Reynolds number equal to 23600 the addition of the excitation is a novel element of the research. There are no experimental results for the excited Cabra flame, and hence, the credibility of the obtained results is proven by comparison with the measurements data available for the non-excited case. The present research is an exploratory numerical study in which we assess large-scale effects of the excitation, i.e., the change of the lift-off height or the change of the size and shape of the flame. It is shown for the first time that the dynamics of the lifted flame can be effectively controlled by a proper choice of the excitation type and its frequency.

## Mathematical approach

We consider a low Mach number reacting flow for which the continuity, Navier-Stokes and transport equations of scalars within the framework of the LES method are defined as:

1 $$\begin{aligned}&\partial _t \bar{\rho }+\nabla \cdot \left( \bar{\rho } \widetilde{\mathbf {u}}\right) =0 \nonumber \\&\bar{\rho }\partial _t\widetilde{\mathbf {u}} +\left( \bar{\rho }\widetilde{\mathbf {u}}\cdot \nabla \right) \widetilde{\mathbf {u}} +\nabla \bar{p}=\nabla \cdot \ \left( \varvec{\tau }+\varvec{\tau }^{SGS}\right) \nonumber \\&\bar{\rho }\partial _t\widetilde{Y}_{\alpha }+\bar{\rho } \widetilde{\mathbf {u}}\cdot \nabla \widetilde{Y}_{\alpha } =\nabla \cdot \left( {\bar{\rho }\left( {D}_{\alpha } +{D}^{SGS}_{\alpha }\right) {\nabla \widetilde{Y}_{\alpha }}}\right) +\overline{\rho \dot{w}}_{\alpha } \nonumber \\&\bar{\rho }\partial _t\widetilde{{h}}+\bar{\rho }\widetilde{\mathbf {u}} \cdot \nabla \widetilde{h}=\nabla \cdot \left( {\bar{\rho } \left( {D}+{D}^{SGS}\right) {\nabla {h}}}\right) \end{aligned}$$

where the bar and tilde symbols denote filtered quantities. The variables in Eqs. (1) are the velocity vector $\mathbf {u}$, the density $\rho$, the hydrodynamic pressure *p*, the species mass fractions $Y_\alpha$ and enthalpy *h*. The subscript $\alpha$ represents the index of the species $\alpha =1,\dots , \text {N-species}$. The quantities $\varvec{\tau }$ and $D_\alpha , D$ are the viscous stress tensor and mass and heat diffusivities. The sub-filter tensor is given by $\varvec{\tau }^{SGS}=2\mu _t\mathbf {S}$, where $\mathbf {S}$ is the rate of strain tensor of the resolved velocity field and $\mu _t$ is the sub-filter viscosity modelled as in^22^. The sub-filter diffusivities in the species and enthalpy transport equations are computed as ${D}^{SGS}=\mu _t/(\bar{\rho }\sigma )$ where $\sigma$ is the turbulent Schmidt or Prandtl number assumed equal to 0.7^23^. The set of equations (1) is complemented with the equation of state $p_0=\overline{\rho } R \widetilde{T}$ with $p_0$ being the constant thermodynamic pressure and *R* is the gas constant.

The chemical source terms $\overline{\rho \dot{w}}_{\alpha }$ represent the net rate of formation and consumption of the species. A highly non-linear nature of this term means that sub-grid fluctuations cannot be ignored. In the present work, the scalar equations (species and enthalpy) are replaced by an equivalent evolution equation for the density-weighted filtered PDF function, which is solved using the stochastic field method proposed by Valiño^21^. Each scalar $\tilde{\phi }_\alpha$ is represented by $1\le n\le N_s$ stochastic fields $\xi _\alpha ^n$ such that $\tilde{\phi }_\alpha =1/N_s\sum _{n=1}^{N_s}\xi _\alpha ^n$. The stochastic fields evolve according to:

2 $$\begin{aligned} \text {d}\xi _\alpha ^n&=-\widetilde{\mathbf {u}}\cdot \nabla \xi _\alpha ^n \text {dt} + \nabla \cdot \left( \Gamma \, \nabla \xi _\alpha ^n\right) \text {dt} +\sqrt{2\Gamma } \, \nabla \xi _\alpha ^n\cdot \text {d}\mathbf {W}\nonumber \\&\quad -0.5\tau _{SGS}^{-1}\left( \xi _\alpha ^n - \tilde{\phi }_\alpha \right) \text {dt}+{\dot{w}}_{\alpha }(\xi _\alpha ^n )\text {dt} \end{aligned}$$

where the total diffusion coefficient is defined as $\Gamma =\left( {D}_{\alpha }+{D}^{SGS}_{\alpha }\right)$, the micro-mixing time scale equals to $\tau _{SGS}={\bar{\rho }\Delta ^2}/({\mu +\mu _t})$ with $\Delta =Vol_{\mathrm{cell}}^{1/3}$ being the LES filter width and $\text {d}\mathbf {W}$ represents a vector of Wiener process increments different for each field. Following Jones and Navarro^24^ eight stochastic fields have been used. The test computations performed with sixteen fields did not show any substantial changes in the dynamics of the flame.

### Numerical method

We apply an in-house numerical code (SAILOR) based on the projection method for the pressure-velocity coupling^25^. The time integration is performed by means of an operator splitting approach where the transport in physical space and chemical terms are solved separately. The convective and diffusive parts of the governing equations are advanced in time using a predictor-corrector technique with the 2nd order Adams-Bashforth / Adams-Moulton methods. The chemical reactions are computed using a CHEMKIN interpreter. In the present study, we analyze the hydrogen combustion using a detailed mechanism of Mueller^26^ involving 9 species and 21 reactions. The reaction terms are stiff and therefore they are integrated in the time applying the VODPK^27^ solver that is well suited for stiff systems. The spatial discretization is performed on half-staggered meshes applying the 6th order compact difference approximation for the Navier-Stokes and continuity equations^25,28^. The convective terms in the stochastic field equations are discretized applying TVD scheme with the van Leer limiter. The applied code has been thoroughly verified in previous studies^19,25,29–31^ and it turned out to be very accurate.

**Figure 1:**
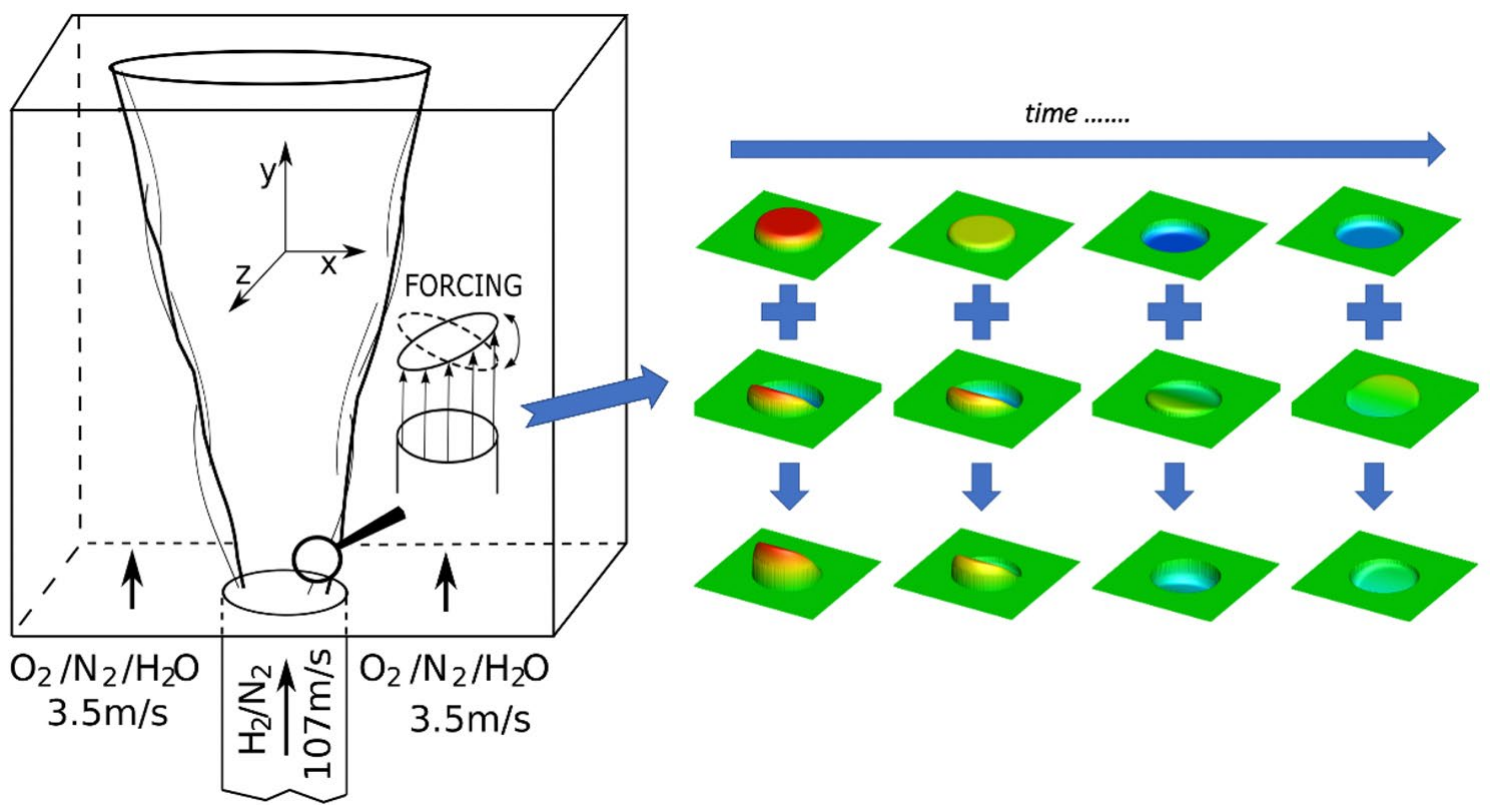
Schematic view of the computational domain with the axial velocity iso-surface showing the time evolution of the axial and flapping forcing.

## Computational configuration

The basic flow configuration analyzed in this study corresponds to the so-called Cabra flame^20^, which we modify by adding the excitation at the nozzle exit. A schematic view of the computational geometry is shown in Fig. 1. It is a rectangular box with dimensions $L_{x}\times L_{y}\times L_{z}=14D\times 30D\times 14D$, where $D=0.00457\,\text {m}$ is the nozzle diameter. The injected fuel ($X_{\mathrm{H}2} = 0.254$, $X_{\mathrm{N}2} = 0.746$) has the temperature $T_{\mathrm{fuel}}=305\,\text {K}$ and the bulk velocity $U_{j}=107\,\text {m/s}$. Outside of the fuel nozzle there is a hot ($T_{\mathrm{cf}}=1045\,\text {K}$) co-flowing stream of the hydrogen combustion products ($X_{\mathrm{O}2} = 0.147$, $X_{\mathrm{H}2\mathrm{O}} = 0.1$, $X_{\mathrm{N}2} = 0.753$) with the velocity $U_{\mathrm{cf}}=3.5\,\text {m/s}$. The excitation (forcing) is introduced as a component of the velocity prescribed at the inlet as $u(\mathbf {x},t)=u_{mean}(\mathbf {x})+u_{turb}(\mathbf {x},t)+u_{excit}(\mathbf {x},t)$, where $u_{mean}(\mathbf {x})$ is the mean velocity profile corresponding to the fully developed pipe flow (1/7 profile) and $u_{turb}(\mathbf {x},t)=0.05U_{j}$ represents turbulent fluctuations computed applying a digital filtering method proposed by Klein et al.^32^. This method guarantees properly correlated velocity fields which reflect realistic turbulent flow conditions. The forcing component $u_{excit}(\mathbf {x},t)$ is added to the streamwise velocity only and it is defined as:

3 $$\begin{aligned} u_{excit}(\mathbf {x},t)=\underbrace{A_a \sin \left( 2\pi f_at\right) }_{\mathrm{Axial}\ \mathrm{forcing}} +\underbrace{A_f \sin \left( 2\pi f_ft\right) \sin \left( \frac{\pi x}{D}\right) }_{\mathrm{Flapping}\ \mathrm{forcing}} \end{aligned}$$

which is the superposition of axial and flapping forcing term with amplitudes $A_a$ and $A_f$ and frequencies $f_a$ and $f_f$. The computations have been performed on three meshes consisting of $120\times 192\times 120$ (coarse), $120\times 264\times 120$ (medium) and $192\times 264\times 192$ (dense) nodes compacted radially towards the flame region and axially towards the fuel nozzle. Preliminary tests showed that the medium mesh provides virtually the same solutions as the ones obtained on the dense mesh. This is due to the high-order discretisation method applied that leads to grid independent results already on relatively coarse meshes. However, to ensure a better resolution of the small scale phenomena the main computations have been performed on the dense mesh. Figure 1 shows a sample temporal evolution of the velocity disturbance when both forcing terms are applied. The Strouhal numbers corresponding to $f_a$ and $f_f$ are defined as $\text {St}_a=f_aD/U_j$ and $\text {St}_f=f_fD/U_j$. In this study we keep the amplitudes constant and equal to $A_a=A_f=0.15U_j$ and we analyze dependence of the flame behaviour on the forcing frequency assuming $\text {St}_a=0.30,\,0.45,\,0.60,\,0.75$ and $\text {St}_f=\text {St}_a/2$ for which the strongest effect of the flapping term was observed^8,19,33^. We consider three possible combinations of the forcing: (i) the axial only - the cases denoted as $A_{\mathrm{St}}$, where the subscript defines the forcing frequency, e.g. $A_{45}$ denotes the axial forcing with $\text {St}_a=0.45$; (ii) the flapping forcing only - the cases $F_{\mathrm{St}}$; (iii) both forcing modes turned on - the cases $AF_{\mathrm{St}}$.

As mentioned in the Introduction section, the simulations and results discussed in this study have an exploratory character as no experiments or numerical data are available for validation of the obtained results regarding the excited flames. Therefore the comparison was performed only based on measurements for the original Cabra flame configuration of which a spatiotemporal complexity is not much different from the cases with the excitation. As will be presented, the agreement between the present solutions and the experimental data is sufficiently good to assume that the obtained results are accurate and reliable.

**Figure 2:**
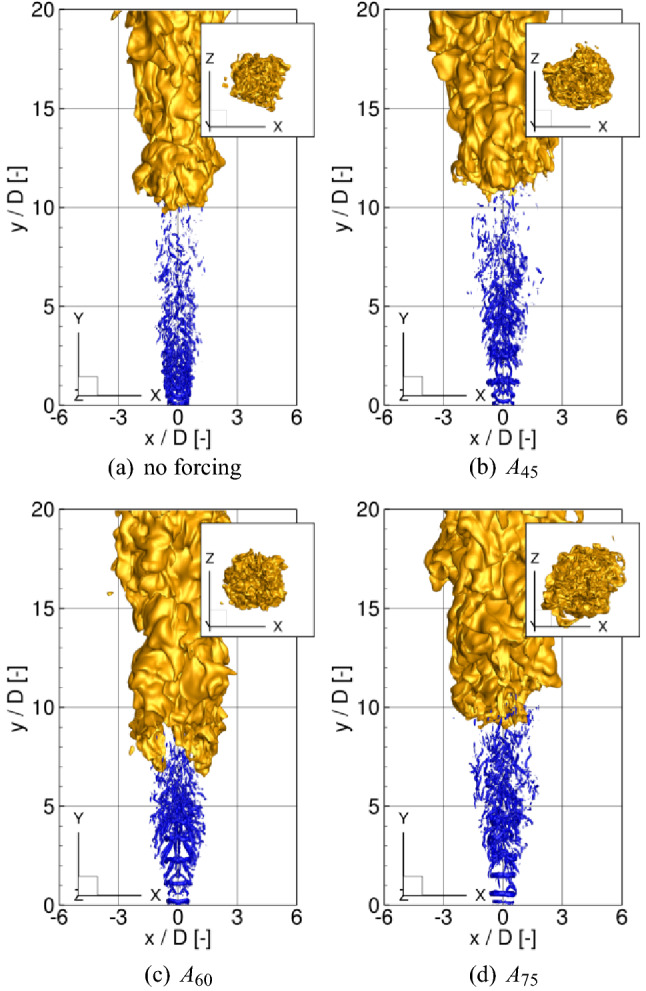
Iso-surfaces of the *Q*-parameter ($Q=10\,\hbox {s}^{-2}$, blue) and temperature $T=1200\,\hbox {K}$ (yellow). Subfigures show the view of the flames from the bottom along the ‘y’ coordinate.

**Figure 3:**
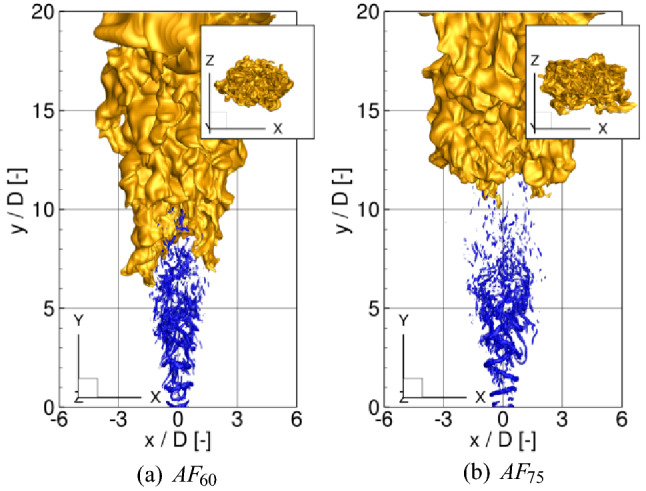
As in Fig. 2 but for $AF_{60}$ and $AF_{75}$.

## Results and discussion

### Three-dimensional flame behavior

The fuel issuing from the nozzle mixes with the co-flowing hot stream and auto-ignites. The ignition spots appear at the locations of the most reactive mixture fraction $\xi _{\mathrm{MR}}=0.053$ at distances far from the inlet, i.e. $y/D\approx 20$. Then, the flame spread radially, propagates upstream and stabilizes as a lifted flame in between $y/D\approx 7.5-11.0$ depending on the test case. Figure 2 shows iso-surfaces of an instantaneous temperature ($T=1200\,\hbox {K}$) and *Q*-parameter ($Q=10\,\hbox {s}^{-2}$) for the cases without the excitation and for $A_{45}$, $A_{60}$ and $A_{75}$. The *Q*-parameter is commonly used to indicate organized vortical motion. It is defined as $Q=1/2(\Omega _{ij}\Omega _{ij}-S_{ij}S_{ij})$, where $S_{ij}$ and $\Omega _{ij}$ are the symmetric and antisymmetric parts of the velocity gradient tensor. Here, it visualizes the effect of the excitation, which manifests by the occurrence of toroidal vortices formed in the vicinity of the nozzle. They mutually interact through rib-like vortices and break up further downstream. Compared to the unexcited case (see Fig. 2a) the vortices are much more pronounced, the distances between them are dependent on the forcing frequency and decrease with increasing $\text {St}_a$. One can notice that in the case $A_{60}$ the jet at $y/D\approx 1.0-4.0$ is wider than for $A_{45}$ and $A_{75}$ and its shape reminds a barrel. The temperature distributions reveal that the flame lift-off height depends on the forcing frequency and turns out to be the smallest for $A_{60}$ that will be further confirmed by time-averaged results. The flame behaviour changes significantly when the flapping forcing is turned on. Figure 3 shows the results for the cases $AF_{60}$ and $AF_{75}$ in which the flapping forcing causes that the toroidal vortices are tilted about the axial direction. Note that this effect is noticeably stronger for $AF_{75}$. For $AF_{60}$ the tilting of the vortical rings is overwhelmed by its very strong amplification by the axial forcing term, as will be discussed in the next sections. In both cases, however, the rings tend to move alternately to the left and right side of the domain and in effect, the flames with the flapping excitation become wider in the ’x’-direction and narrower in the ’z’-direction. In Tyliszczak A.^19^ it was shown that for a low Reynolds number ($\text {Re}=4000$) the flame can even bifurcate (i.e. split into two separate branches), however, this phenomenon does not occur here. For the present case with $\text {Re}=23600$ and a relatively high level of the inlet turbulence intensity the toroidal structures are destroyed before reaching a bifurcation point that usually is located at $y/D\approx 5$^33^.

**Figure 4:**
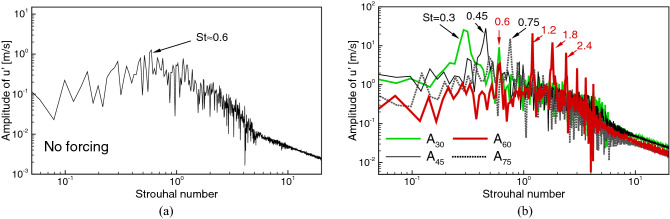
Velocity spectra at $y/D=3.0$ without forcing (**a**) and with the axial forcing (**b**).

### Effect of the excitation in spectral space

Figure 4a shows an axial velocity spectrum computed based on a velocity signal recorded in the axis at $y/D=3.0$ for the case without the excitation. It can be seen that there are no distinct peaks in the spectrum that could be related to the preferred mode frequency or the parring process. Apparently, as can be seen in Fig. 2a, the level of turbulence imposed at the inlet prevents the formation of strong, well defined vortical structures of which periodic occurrence would certainly manifest also in the spectrum. Instead, only a rise of the amplitudes of the fluctuations at a broadband range of the frequencies is observed, which is centred around $\text {St}=0.6$. The excitation at $\text {St}_a=0.60$ was chosen to match exactly this value, whereas the excitation at $\text {St}_a=0.30$ corresponds to its sub-harmonic at which the parring process could exist. Figure 4b shows the velocity spectra for the cases with the axial forcing only. Distinct peaks corresponding to the excitation frequencies are readily seen as they are pronouncedly larger than the background level. The cases $A_{45}$ and $A_{75}$ do not show anything exceptional. The peaks related to their basic frequencies are virtually the only ones visible. Further downstream they become wider and lower (not presented) and it seems that there are no additional phenomena created by the forcing at these frequencies. The results for $A_{30}$ and $A_{60}$ are significantly different. In the former case (green line in Fig. 4b) the excitation causes intensified velocity fluctuations not only at the basic frequency but also at its harmonic $\text {St}=0.60$. The most striking difference is, however, for the case $A_{60}$ for which the whole bench of highly energetic harmonics is found. They appear as the results of interactions between subsequent vortices. These interactions take place through the elongated rib structures (see Fig. 2c) that connect the vortical rings. One could expect that existence of harmonics causes an intensified mixing at small scales that speeds up the ignition process. The velocity spectra obtained for the cases with the axial and flapping forcing acting together show similar features, though, the harmonics are much weaker. The spectra for the cases with the only flapping excitation turned on are not significantly different from the ’no forcing’ case as in the axis locations close to the inlet the impact of the flapping should not be pronounced by definition.

**Figure 5:**
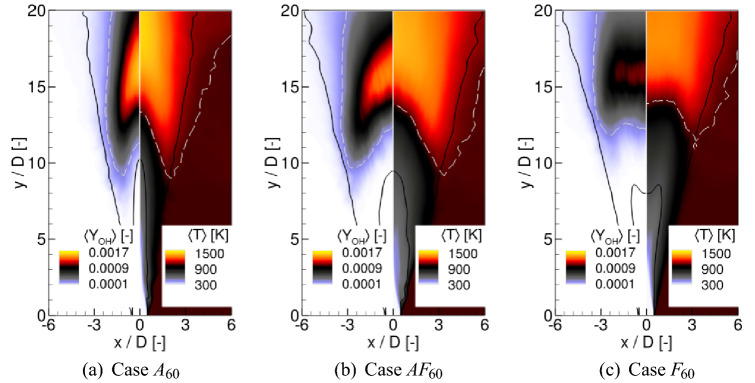
Contours of the time averaged OH mass fraction and temperature in the central ‘x-y’ cross-section plane.

**Figure 6:**
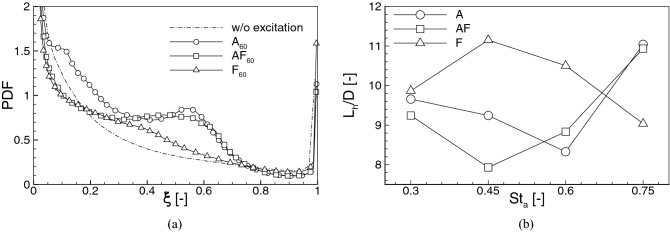
PDF of the mixture fraction (**a**) and the lift-off height of the flames (**b**).

**Figure 7:**
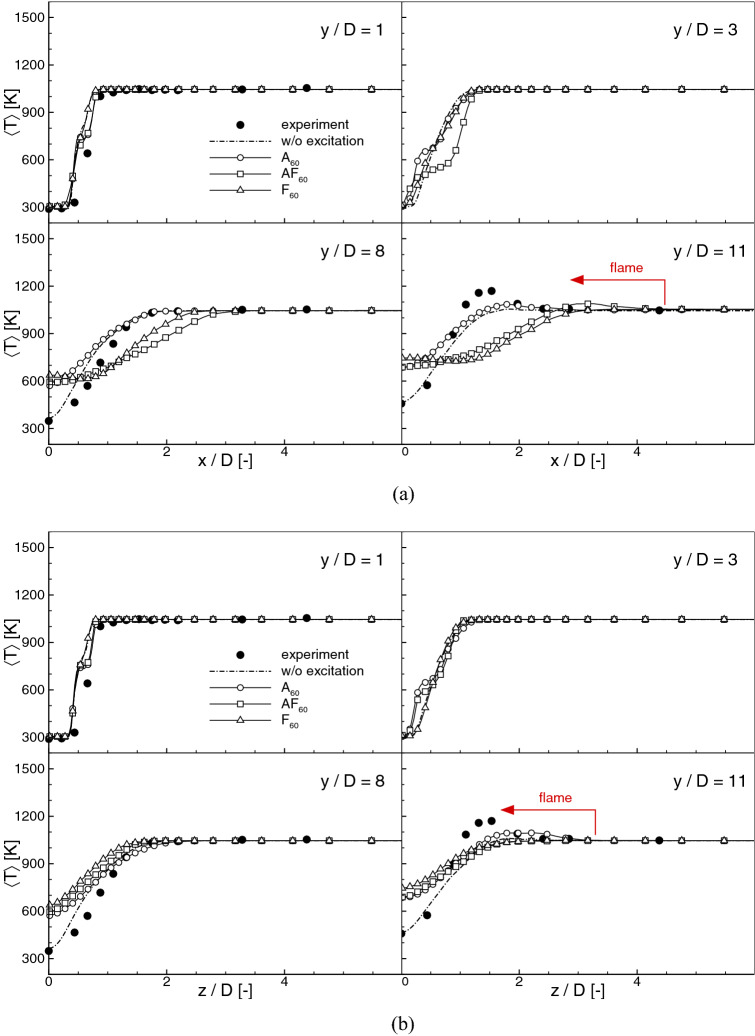
Meant temperature profiles for $\text {St}_a=0.60$ along the ‘x’-direction (**a**) and the ‘z’-direction (**b**).

**Figure 8:**
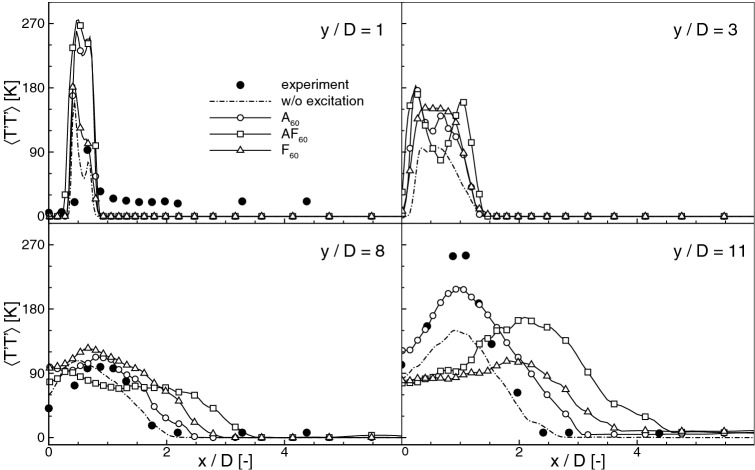
Temperature fluctuation profiles for $\text {St}_a=0.60$.

### Impact of the excitation on the lift-off height

The lift-off height ($L_h$) of the flames is estimated based on the time-averaged results. The time-averaging procedure started when the flames were fully developed and it lasted for a period of at least 300*D*/*U*, which was found sufficient to obtain well convergent statistics. Figure 5 shows the contours of the time-averaged OH mass fraction and temperature in the central ‘x-y’ cross-section plane. The inner black lines represent the stoichiometric mixture fraction $\xi _{\mathrm{ST}}=0.476$ and the outer ones correspond to $\xi _{\mathrm{MR}}$. The white dashed lines denotes the OH mass fraction equal to $Y_{\mathrm{OH}}=2.0\times 10^{-4}$ and $T=1.01T_{\mathrm{cf}}$, which are the typical criteria used to estimate $L_h$^34^. It can be seen that both the shapes of the flames and $L_h$ are dependent on the type of the excitation. The $L_h$ was measured as the lowest point in the domain where the temperature or $Y_{\mathrm{OH}}$ exceeded the above-defined thresholds. In all the cases these points occur not in the flame axis but a few diameters off-axis. The $L_h$ predicted based on the temperature criterion is slightly smaller than using the OH mass fraction criterion, however, the differences are not very significant ($\Delta L_h< 1.0D$). Worth noting is that both threshold lines predict very similar behaviour. In case $A_{60}$ in the central part of the flame these lines are almost straight and their inclinations to the flame axis depend on the forcing frequencies (not shown). When the flapping forcing is turned on the threshold lines become rounded. Figure 6 shows the PDF of the mixture fraction in the shear layer region (box of $5D\times 1.5D \times 1.5D$ located 2*D* below the flame front) for the cases with $St_a=60$, and the dependence of the $L_h$ on the forcing frequencies for all analyzed configurations. A significant increase of the PDF around $\xi _{\mathrm{ST}}=0.476$ for $A_{60}$ and $AF_{60}$ compared to the case $F_{60}$ and without the excitation means an intensified mixing process that should manifest by a shortening of $L_h$. Indeed, for the case without the excitation $L_h=9.7D$ ($L_h=10D$ in the experiment^20^), which is visibly different from $L_h$ found when the excitation is applied. In these cases, depending on the forcing frequency and excitation type $L_h$ changes in the range $7.9D-11.2D$. Its lowest value occurs when the axial and flapping excitation act together. Turning on only the flapping mode causes that $L_h$ reaches the maximum at $St_a=0.45$ after which it continuously decreases as the effect of intensified mixing of smaller spatiotemporal flow scales caused by higher forcing frequencies. For the axial excitation, both acting solely or together with the flapping mode, $L_h$ behaves differently. It reaches the maximum at $St_a=0.75$, but first, i.e., for $St_a<0.75$, it suddenly drops down for $St_a=0.45$ and $St_a=0.60$ for the axial-flapping and axial modes, respectively. The occurrence of these minima can be to some extent related to the appearance of the high-frequency harmonics in the spectra in Fig. 4b. They have a similar impact on the mixing as the increase of the forcing frequency of the flapping excitation. The enhanced mixing for the flapping excitation mode at higher $St_a=0.75$ is mainly due to an induced radial mixing of the fuel with the hot co-flow. This is not observed for the cases with the axial and axial-flapping excitations. In these configurations, the high frequency of the axial fuel oscillation seems to prevail over the radial motion. Most likely because of the interaction of the excitation with naturally induced vortices.

### Impact of the excitation on time-averaged results

It could be observed in Fig. 5 that the excitation changes not only the flames positions but also visibly influences their shapes and spreading angles. Compared to the cases with the axial excitation, the flapping forcing makes the flame significantly wider. Figure 7 shows the profiles of time-averaged temperature along the ‘x’ and ‘z’ directions compared with the experimental data at $y/D=1,3,8,11$, and in Fig. 8 the profiles of the temperature fluctuations along the ‘x’ direction are presented. First, it should be noted that the present results agree quite well with the measurements. The location where the fuel burns in the shear layer ($y/D\approx 11$) and the near axis temperature distributions are correctly captured. Worth noticing is the fact that in the experiment the co-flow temperature was biased by the 3% error^20^ that could lead to $\pm 15\,\hbox {K}$ difference compared to the assumed $T_{\mathrm{cf}}=1045\,\hbox {K}$. As observed by Navarro-Martinez and Kronenburg^35^ the co-flow temperature has a substantial impact on the flame stabilization height. Nevertheless, it seems that $\pm 15\,\hbox {K}$ error in $T_{\mathrm{cf}}$ has not a significant impact on the present results. Regarding the excited flames it can be readily seen that close to the inlet all profiles (also for the non-excitated case) are very similar and differences start to be seen only downstream. The temperatures are definitively larger in the axis for all excited cases, whereas for the cases *AF* their maxima move radially towards larger *x*/*D* locations. In general, the axial excitation causes a faster temperature rise, while the flapping forcing makes the temperature more uniform along the ‘x’-direction. The profiles along the ‘z’-direction (Fig. 7b) show that further from the nozzle the flapping excitation can lead to 30% narrowing of the flame in respect to the ‘x’-direction. Taking into account the fluctuations profiles one can see that the excitation rises their level close to the nozzle. Further downstream and in the vicinity of $L_h$ location the flapping mode flattens the profiles and shifts their maxima in the ‘x’-direction. This is the effect of a more intense mixing with the co-flowing stream that makes the excited flames wider.

## Conclusions

The paper presented the LES studies of the $\text {H}_2/\text {N}_2$ flame excited with the axial and flapping forcing. The correctness of the results was confirmed by comparison with available experimental data for the unexcited case. It was found that the lift-off height of the flame, its size and shape can be altered in a wide range depending on the type of the excitation and its frequency. Compared to the unexcited flame the lift-off height can be increased or decreased. Its minimum value has been found for the case with the combination of both the axial and flapping forcing at the frequency close to the centre of the broadband frequency range regarded as the preferred one in the non-excited configuration. The impact of the flapping forcing manifested through a widening of the flame in the flapping direction.
